# Talks about Food

**Published:** 1891-10-17

**Authors:** 


### Talks about Food.

II.—THE RELATION OF FOOD TO LABOUR (*Continued*).

Demography is quite the fashion just now, and we rather expect to see all persons who are interested in bettering the condition of the people, endeavouring to make out that their special line or department is but a branch of this fashionable subject—as indeed they may very fairly do, if we look only to the derivation of the word. Our present subject, however, cannot be said to need dragging in by the heels at all, so manifest is it that health and well-being are simply impossible unless considerable attention is paid to the food question. In our last article we pointed out that it has an important bearing upon our success, or non-success, in competing with foreign workpeople; and it may also be remarked that, when we are studying that other great problem of the day, the decline of the agricultural population and the almost alarming growth of our large towns, it will be well to keep the food question in sight. The agricultural population is declining, we fear, not only in numbers, but in physique and morale; and to our thinking there are two reasons for this. One is that the best and most enterprising young men are drawn away to the towns by the prospect of higher wages and a less colourless life, thus leaving the country to be peopled by a gradually deteriorating race; the other is that country wages being very low, and the labourer's knowledge of what constitutes a nutritive diet but small, he is insufficiently nourished. In all parts of the country you may hear the farmers complain that their labourers are not worth even the wages given them, and that there was a very different set of men on the land even in their young days; and although we must allow something for the farmers traditional habit of grumbling, we believe that there is a considerable amount of truth in his complaint.

Whether it really pays him, however, to give his labourers such low wages is a matter upon which we feel some doubt. There is a well-known story of a man who, when asked how it was that he paid his labourers so well, replied that he "could not afford to pay them less." And, as a standard writer on food and dietetics has remarked, "one might just as reasonably expect that a fire would burn briskly with a scanty supply of fuel, or a steam-engine work with a deficient supply of coal, as that a man will labour upon a meagre diet." Put "horse" instead of "man," and this proposition will receive instant assent from every one—we suppose because, with the former, effect follows immediately upon cause, a feed of oats brisking up a horse almost instantly; but with regard to the latter, it seems to be often lost sight of. Doubtless the farmers will claim to know their own business best; but to the plain man it seems bad policy, and bad sense too, to give a labourer wages which can only command a scanty diet, and then complain because you cannot take out of him what you never put in; and we should like to see this pressed home upon the farmers. Doubtless, too, they would answer that better wages only mean more frequent and protracted visits to the public-house; and we confess to having ourselves noticed that drinking and wages go up and down together. But what applies to a temporary rise in wages does not, we believe, apply to a permanent one—an opinion in which we are supported by others who have long worked among the poor.

This is not merely a question of wages, however. As we pointed out in our first article, increased efficiency need not mean increased cost; and it is by no means hopeless (as we shall hope to show later on) to improve the diet of our country people, even if their wages remain the same.

Moreover, it should always be remembered that, if we benefit the country in this way, we shall also benefit the towns. Mr. Charles Booth has given us a graphic description of the way in which the country becomes a "feeder" for the great towns, bringing up year by year its awkward squads of raw, bucolic youths, who, nevertheless, by virtue of their finer physique and greater bodily strength, oust the town-born man from all employments requiring a brawny arm and a deep chest. Seeing, then, that our big towns act as "exhausters" (and anyone who doubts this should read Dr. Milner Fothergill's "Town Dweller"), it becomes of the greatest importance to secure, if possible, that the fresh supplies to which we must look shall be of the best quality. The feeder must be fed, and fed intelligently and adequately. Fresh air and good food—these are the requisites for a healthy life and a burly frame. The former our country labourer has; the latter we must endeavour to supply.

But we have been speaking hitherto as if the question of food were of importance to the poor alone, or, at any rate, only indirectly—inasmuch as everything which affects one class must needs affect another—to the rich. This, however, is by no means the case. True, it is of less importance to us than it is to the poor to extract the full value of, say, a peck of wheat, because if we choose to throw away a nutritious part of it, we can make up for the loss by eating something else; neither are we in much danger of losing in force and vigour through deficiency of proper food. But waste is always obnoxious, even when a man's purse is full to overflowing; and as for dangers—hear that well-known authority on the food question, Dr. Pavy: "There is far more evil to be encountered attributable to too much food being taken than to too little. It is only in exceptional cases that the latter kind is met with; whilst the amount of disorder, disease, and likewise even curtailment of life, attributable to excess in eating and drinking, is immeasurably great."

It is much to be wished that mothers of families, heads of schools, and indeed all intelligent persons, would study this subject—a really simple one, as far as practical knowledge is concerned. It would be far better than dabbling in drugs, as so many ladies are apt to do; and it would save the expense of a doctor quite as much—nay, more. Unfortunately, we—that is the non-medical public—have left this subject far too much to the faddists – to people who talk of meat as "dead flesh," or "corpses," who glare at a loaf of white bread like a bull at a red rag, who run wild on the sin of over-eating, and preach abstinence as though it were the only means of grace. We may well ask, as did a weekly contemporary some little time ago, "Where are the anti-faddists?" They might do good work if they would take up this subject reasonably, dispassionately, and thoughtfully.

---

Mr. Robert Louis Stevenson is quoted as stating that, "there are men and classes of men that stand above the common herd, the physician almost as a rule"; for which doctors of course are duly grateful. It has also been stated of him recently, that he "is *ridiculously* well," of which we are very glad to hear, though we don't quite know what it means. This remarkable state of health must be embarrassing to him in his more serious moments.

The press of New York has recently been exercising its mind upon the questing whether medical women should marry or not. The *New York Medical Record* holds that they should not, owing to the exacting nature of a physician's work, and would confine marriage only to such medical women as devote themselves to some narrow speciality which makes only short and specific demands upon their time. This is rather hard upon medical women, for their nature cannot differ from that of trained nurses, who, in this country at least, look upon marriage with no jaundiced eye.

